# Multiple markers of cortical morphology reveal evidence of supragranular thinning in schizophrenia

**DOI:** 10.1038/tp.2016.43

**Published:** 2016-04-12

**Authors:** K Wagstyl, L Ronan, K J Whitaker, I M Goodyer, N Roberts, T J Crow, P C Fletcher

**Affiliations:** 1 Brain Mapping Unit, Department of Psychiatry, University of Cambridge, Cambridge, UK; 2 Developmental and Life-course Research Group, Department of Psychiatry, University of Cambridge, Cambridge, UK; 3 Clinical Research Imaging Centre, School of Clinical Sciences, University of Edinburgh, Edinburgh, UK; 4 Department of Psychiatry, Warneford Hospital, University of Oxford, Oxford, UK; 5 Cambridge and Peterborough Foundation Trust, Cambridge, UK

## Abstract

*In vivo* structural neuroimaging can reliably identify changes to cortical morphology and its regional variation but cannot yet relate these changes to specific cortical layers. We propose, however, that by synthesizing principles of cortical organization, including relative contributions of different layers to sulcal and gyral thickness, regional patterns of variation in thickness of different layers across the cortical sheet and profiles of layer variation across functional hierarchies, it is possible to develop indirect morphological measures as markers of more specific cytoarchitectural changes. We developed four indirect measures sensitive to changes specifically occurring in supragranular cortical layers, and applied these to test the hypothesis that supragranular layers are disproportionately affected in schizophrenia. Our findings from the four different measures converge to indicate a predominance of supragranular thinning in schizophrenia, independent of medication and illness duration. We propose that these indirect measures offer novel ways of identifying layer-specific cortical changes, offering complementary *in vivo* observations to existing post-mortem studies.

## Introduction

The cerebral cortex has a predictably varying laminar structure.^1^ Individual layers exhibit differing histological composition, regional distributions,^2^ developmental trajectories,^3^ physiology^4^ and hypothesized functional roles.^5^ The question arises whether a subtle variation in these components could contribute to the spectrum of psychiatric syndromes including schizophrenia.^6^ However, direct identification of these putative lamina changes is necessarily limited to post-mortem analysis given that structural magnetic resonance imaging (MRI) methods currently cannot resolve individual cortical layers *in vivo*.

Accumulating evidence from several approaches has indicated that there are alterations in the supragranular layers in schizophrenia.^6^ These include deficits in small interneurons,^7^ reduced density of calbindin cells in layer II,^8^ as well as changes in neurotransmitters, receptors,^9^ pyramidal cell density and morphology,^10, 11^ and mRNA expression^12^ in layers II and III. In addition, supragranular thinning has been measured in the dorsolateral prefrontal cortex (DLPFC/BA46)^13^ although the extent of these changes across the cortex has not been established. Although there is some heterogeneity reported in the neuropathology of schizophrenia,^14^ supragranular layer changes appear to be a consistent finding and are not caused by prolonged exposure to antipsychotic medication^15^ or alcohol abuse.^16^ *In vivo* studies of cortical structure in schizophrenia have identified regional changes in cortical thickness^17^ and surface area,^18^ but limits to MRI resolution mean that direct markers of supragranular cortical change are not currently available.

We attempted to address this problem indirectly by capitalizing on principles of structural brain organization that potentially differentiate between alterations in the infragranular (V and VI) and supragranular (I–III) layers of the cortex *in vivo*. Specifically, we identified four measures that are relatively specific to supragranular change and used these measures to evaluate a previously acquired data set^19^ from people with schizophrenia, in order to determine whether measurable neuroanatomical changes favored the prediction that cortical pathology in schizophrenia is predominant in supragranular layers. The four measures are explained below.

**Figure 1:**
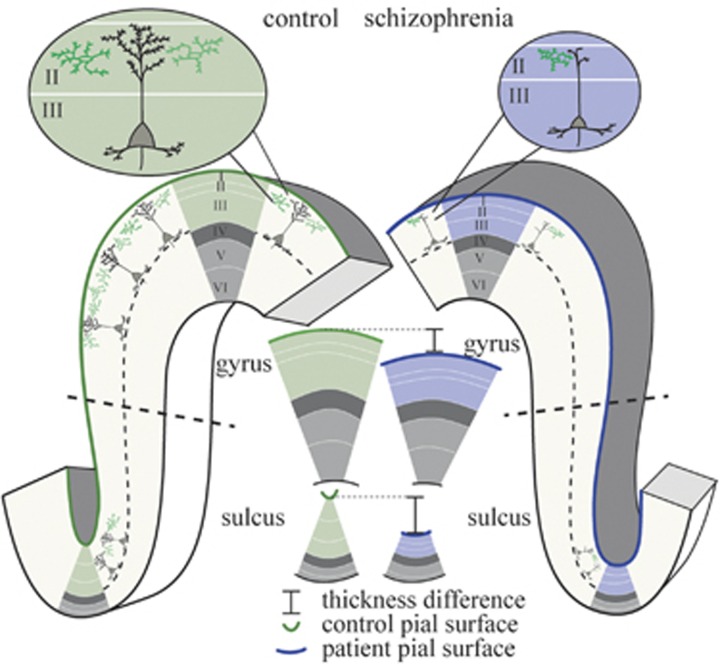
Morphological effect of disproportionate supragranular thinning (I–III, green/blue; infragranular, V and VI, light gray). Decreased dendrites and synapses in schizophrenia can result in thinner supragranular layers, which may in turn be reflected in changes to large-scale cortical morphology. In particular, supragranular layers are thicker in sulci than in gyri, thus pruning will lead to disproportionately reduced cortical thickness in sulci compared with gyri. Similarly, pruning of the supragranular layers will affect the curvature of the pial surface more than the curvature of the boundary between gray and white matters, again disproportionately more in sulci than gyri.

First, the thicknesses of supra- and infragranular layers are consistently different between the crowns of gyri (where infragranular layers are thicker) and the depths of sulci (where supragranular layers are thicker) (Figure 1).^2, 20, 21, 22^ This is a product of deformation of the cortex under folding, such that layers that are on the outside of a fold (lower layers in sulci and upper layers in gyri) are stretched and thinner, whereas layers on the inside of folds are compressed and thicker.^20^ For example, in the prefrontal cortex, the supragranular layers make up 70% of the thickness of a sulcal fundus but only 49% of the adjacent gyral crown.^2^ Thus, despite sulci being generally thinner than gyri,^2, 23^ supragranular layers have an increased relative and absolute thickness in sulci. It follows from these clear folding-related differences in sulci and gyri that supragranular thinning will affect sulci disproportionately. This was the first hypothesis tested here. Specifically, if neuropathology in schizophrenia results in a disproportionate reduction in supragranular layer thickness, we would expect to measure disproportionate thinning of sulci, particularly in regions where these layers are more prominent. This is a general principle that may be applied to the whole brain or to individual lobes and brain regions.

Second, turning to small-scale (but quantifiable) measures of cortical morphometry in the form of intrinsic curvature,^24^ we predict that these will also vary with supragranular layer changes.^19^ Specifically, changes observed superficially (on the pial surface) could feasibly be related to thinning of superficial layers, whereas those observed on the white matter surface could more feasibly be related to thinning in deeper layers. Moreover, in line with the reasoning outlined above, we expect these changes to be more prominent in sulci than gyri.

Third, it is known that cytoarchitecture is not uniform across the healthy human cortex^1^—and that layer thicknesses exhibit significant and consistent variability.^2, 25^ One clear variation in regional patterns of supragranular thickness can be seen across DLPFC/BA46 (where it tends to be thicker) and the anterior cingulate/BA24 cortex (where it is particularly thin). We therefore hypothesized that supragranular thickness alterations in pathological cases may be more prominent in DLPFC, where these layers are thicker, than in anterior cingulate cortex, for example, where these layers are thinner. An extension of this hypothesis is that the pattern of sulcal-specific thinning should follow supragranular layer thicknesses in healthy individuals.^2^

Finally, as well as gyral–sulcal differences, gradients of supragranular (I–III/IV) layer thickness exist within sensory hierarchies. In particular, sensory hierarchies have a gradient of absolute cortical thickness, with thinner primary sensory regions and progressively thicker higher cortical regions.^26^ Supragranular layers are the origin of feedforward connections, communicating incoming sensory information. They are relatively thicker in these lower sensory regions.^2, 25^ By contrast infragranular, feedback layers (V and VI) are more prominent in higher sensory and frontal cortical areas. Therefore, a loss of supragranular thickness should result in a relatively steeper gradient of cortical thickness across the structural hierarchy of sensory systems (that is, preferential reduction of cortical thickness in lower sensory regions).

In summary, we aimed to capitalize on basic biological observations of the natural variation in supragranular thickness in order to relate alterations in magnetic resonance-based measurements of cortical thickness and curvature to underlying supragranular changes. In order to demonstrate the applicability of these measures, we applied them in a schizophrenia case–control cohort.

Our main objective was therefore to quantify supragranular-specific differences in cortical structure measured on structural T1-weighted MRI scans of patients with schizophrenia. We predicted that the cortex in schizophrenia would not only be thinner in line with previous magnetic resonance-based and neuropathological studies, but that disproportionate changes to the upper cortical layers would, on the basis of the above principles, cause thinning to be disproportionate in sulci (which have a greater supragranular thickness) and lead to abnormal curvature of the pial, but not the white matter surfaces, particularly in sulci. In addition, the principles lead us to hypothesize that disproportionate sulcal thinning would be greater in regions with thicker supragranular layers such as BA46 but not in anterior cingulate cortex. Finally, we predicted that the gradient of thickness in the visual hierarchy would be steeper in patients with schizophrenia. These four measures taken together may be adopted as macroscale surrogate markers of changes not accessible with current MRI measures.

## Materials and methods

Forty-six patients (36 males; 33.2±9 years) were recruited by collaborating psychiatrists from Oxfordshire and Berkshire Mental Healthcare Trusts, and with the guidance of the Oxford and Berkshire Psychiatric Research Ethics Committees, UK. Diagnosis was confirmed using the Structural Clinical Interview for DSM-IV Disorders.^27^ Forty-four controls (32 males) were also recruited (30.4±8 years). There were no statistically significant differences in age or sex (*P*>0.05) between patients and controls. Full Scale Intelligence Quotient (FSIQ) was available for 32 controls (122.4±15.3) and 28 patients (101.8±20.4), and was significantly reduced in the patient group (*B*=−20.7, *t*=−4.46, *P*<0.0001). Medication and dose were available for 31 patients from which were derived chlorpromazine equivalent doses (417.5±296.7mg daily); duration of illness was calculated for 32 patients (121.3±87.6 months). Positive and Negative Syndrome Scale (PANSS) was available for 30 patients (93.77±20.3).

Structural MRI data were acquired using a 1.5-T Sonata MRI system (Siemens Medical Systems, Erlangen, Germany) with a standard quadrature head coil and maximum 40 mT m^−1^ gradient capability at the Oxford Centre for Clinical Magnetic Resonance Research. Whole-brain T1-weighted images were acquired with a FLASH sequence using the following parameters: coronal orientation; image matrix=256 × 256, with 1 × 1 mm^2^ in-plane resolution; 208 slices of slice thickness 1 mm; echo time (TE) =5.6 ms; repetition time (TR) =12 ms; and flip angle *α*=19°.

### Cortical reconstruction and analysis

Cortical reconstructions were generated using the software FreeSurfer 5.2 (freely available from http://surfer.nmr.mgh.harvard.edu/).^23, 28, 29^ In brief, raw image data voxels were sub-sampled to voxels of side 1 mm^3^. The data were then normalized for intensity, radio-frequency-bias field inhomogeneities were modeled and removed, followed by skull-stripping. The cerebral white matter was subsequently identified after which the hemispheres were separated, tessellated and deformed to produce an accurate and smooth representation of the gray–white interface. These surface reconstruction processes were conducted in native space. To correct for minor inaccuracies, the reconstructions were manually edited. Eight scans (three patients and five controls) were omitted from further analyses due to large errors or artefacts. Mean curvature was measured to divide the cortex into gyri and sulci. Gyri have a negative mean curvature; sulci have a positive mean curvature.

### Morphometric measurements of supragranular layer thickness changes

Four distinct morphometric markers of supragranular thickness changes were developed, which are explained below.

#### Whole-brain gyral–sulcal thickness differences

Cortical thickness was measured as the shortest distance between each vertex on the white matter surface and the pial surface.^23^ Mean gyral and sulcal thicknesses were calculated for each individual, as was the difference between these two measures. The ratio between total gyral and sulcal surface area was also calculated to test for systematic changes in cortical surface classification.

#### Whole-brain gyral–sulcal intrinsic curvature differences

Intrinsic or Gaussian curvature was calculated for each vertex on the cortex on the white matter and pial surface reconstructions as the product of the principal curvatures.^19, 24^ Mean modulus of intrinsic curvature was calculated for gyral and sulcal cortex at the white matter and pial surfaces, along with the difference between gyral and sulcal measurements.

#### Regional specific pattern

A local measure of gyral–sulcal thickness differences (GSDs) was created as a normalized difference between mean gyral and sulcal cortical thickness within a 25-mm radius of each vertex *i* on an inflated cortical surface.


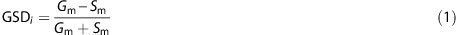


The value of this measure increases when GSDs increase. Thus, an increase in GSD is taken as a measure of the extent to which thinning is sulcal-specific. A unit of 25 mm was chosen as the disk radius to balance local specificity and capturing sufficient gyral and sulcal cortex, independent of central vertex location.^30^ Supplementary Figure 1 shows the effect of varying disk radius on the relative areas of gyral and sulcal cortex captured by the disk.

Per-vertex GSD was registered from individuals to an average surface, and comparison was carried out between patients and controls, controlling for differences in white matter total surface area. Total brain surface area is related to other morphometric measures such as cortical thickness and therefore was taken into account in the regression model.^31^

Furthermore, in order to assess the specificity of our gyral–sulcal-derived markers of supragranular thinning, we compared our regional measure of sulcal-specific thinning with previously reported post-mortem findings. Thickness measurements for cortical regions were taken from von Economo.^2^ Where explicit measurement for a layer was omitted, approximate layer thicknesses were inferred based on textual description, annotated figures and comparison with measurements from the remaining five layers. These values were compared with regional measures of sulcal-specific thinning in schizophrenia using the population-average, landmark- and surface-based (PALS) atlases of Brodmann areas.^32, 33^ Von Economo regions were identified based on the Brodmann atlas and reference tables.^34^

#### Cortical thickness gradient in visual hierarchy

Cortical thickness gradients across the visual hierarchy were calculated for all subjects.^26^ Briefly, visual regions were parcellated on individual subjects according to the PALS visuotopic atlas.^32, 33^ These regions were given estimates of hierarchical level derived from functional studies.^35^ The gradient of regional cortical thickness against visual hierarchical position was calculated using a linear model. Gradients were then compared between patients and controls, accounting for hemispheric differences.

### Statistical analyses

Statistical analyses of the data were carried out using MATLAB^36^ and R.^37^ Patient–control differences were calculated for each of these measures using a linear mixed effects model, controlling for hemispheric differences, total surface area, age, sex and the random effect of individual. The effect of FSIQ on measures 1, 2 and 4 was calculated on the 60 individuals for whom the measure was available, accounting for hemispheric differences, total surface area, sex and the random effect of individual. The effect of PANSS, medication and illness duration on measures 1, 2 and 4 were calculated within the patient group with a linear mixed effects model accounting for hemispheric differences, total surface area, sex and the random effect of individual.

## Results

### Whole-brain GSDs

**Figure 2:**
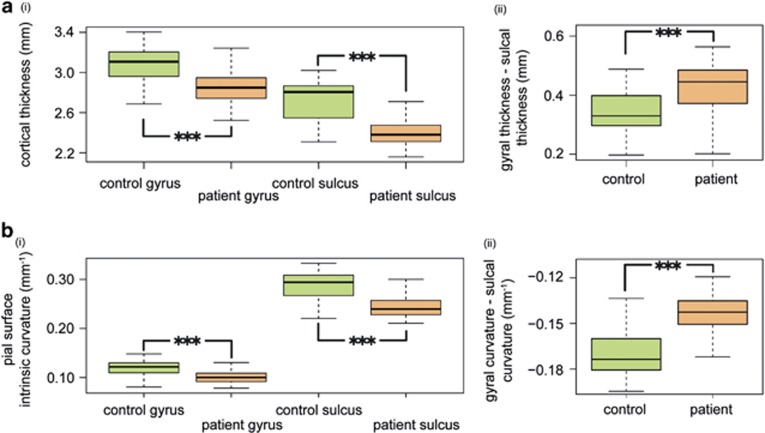
(**a**) (i) Cortical thickness is decreased in both gyri and sulci in schizophrenia (*P*<0.0001). (ii) Supragranular layers are thicker in sulci so that supragranular pathology in schizophrenia leads to a disproportionate decrease in the thickness of the cortex in sulci compared with gyri (*P*<0.0001). (**b**) (i) Intrinsic curvature at the pial surface is decreased in both gyri and sulci in schizophrenia (*P*<0.0001). Consistent with a predominantly upper cortical layer change, there was no difference in intrinsic curvature at the white matter surface. (ii) For the same reason as in **a** (ii) above, sulcal intrinsic curvature is disproportionately decreased relative to gyral curvature in schizophrenia (*P*<0.0001).

In line with neuropathology and previous neuroimaging studies, cortical thickness was significantly decreased in patients with schizophrenia in both gyri (*B*=−0.21, *t*=−6.20, *P*<0.0001) and sulci (*B*=−0.29, *t*=−7.12, *P*<0.0001; Figures 1 and 2a(i); see Supplementary Figure 2 for vertex-wise cortical thickness differences). The difference between mean gyral and sulcal thickness was greater in patients with schizophrenia than in controls (*B*=0.08, *t*=4.60, *P*<0.0001; Figure 2a(ii)). This is in line with the hypothesis that supragranular pathology would be differentially expressed in sulci, owing to the relatively increased supragranular thickness in these regions. There were no significant differences in the ratio of gyral/sulcal cortical surface area (*P*=0.39), thus increased GSD was not caused by systematic misclassification of the cortical surface. There was a small effect of FSIQ on GSD (*B*=0.0012, *t*=2.06, *P*<0.05), but no effect of sex or age. There was no significant effect of PANSS (*P*=0.12), medication (*P*=0.12) or illness duration (*P*=0.14) on GSDs within the patient group.

### Intrinsic curvature

Consistent with upper cortical layer changes driven by supragranular pathology, there was a significant reduction in pial surface intrinsic curvature of both gyri (*B*=−0.017, *t*=−5.59, *P*<0.0001) and sulci (*B*=−0.040, *t*=−6.97, *P*<0.0001; Figure 2b(ii)), but no change in curvature of the white matter surface intrinsic curvature of gyri (*B*=0.001, *t*=0.29, *P*=0.77) or sulci (*B*=0.001, *t*=0.44, *P*=0.66).

Moreover, given the increased prominence of upper cortical layers in sulci, the sulcal curvature was disproportionately decreased, relative to gyral curvature in subjects with schizophrenia (*B*=0.023, *t*=7.21, *P*<0.0001; Figure 2b(ii)). There was no effect of sex, age or FSIQ on gyral–sulcal pial curvature difference. There was also no effect of PANSS (*P*=0.89), medication (*P*=0.92) or illness duration (*P*=0.22) on gyral–sulcal curvature differences within the patient group.

### Regional specific pattern

**Figure 3:**
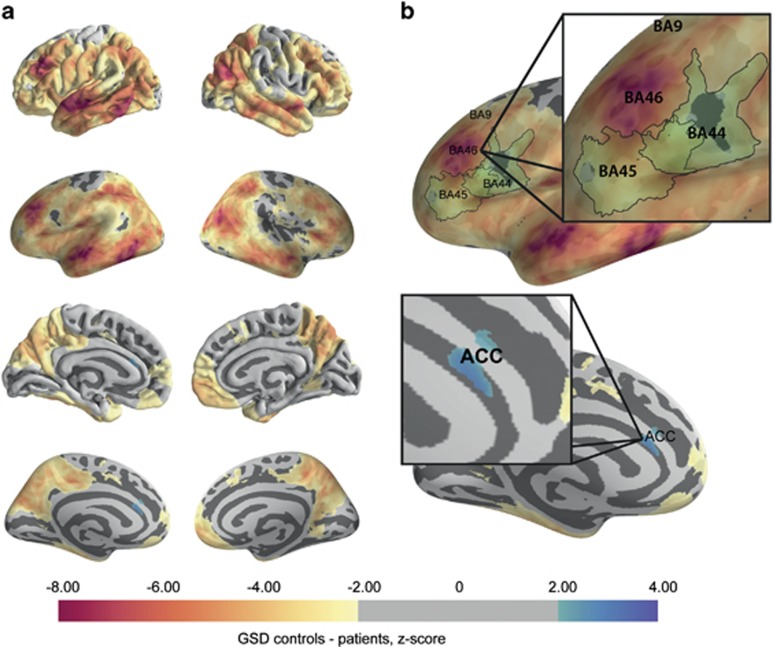
Per-vertex comparison of gyral–sulcal thickness differences (GSDs). (**a**) Local GSDs are regionally increased particularly in dorsolateral prefrontal cortex (DLPFC/BA46), superior temporal gyrus, inferior temporal gyrus and inferior parietal gyrus. (**b**) The regional pattern of sulcal-specific thinning is consistent with neuropathology studies of schizophrenia, which have identified layer II thinning in BA46,^13^ but not BA9, BA44 (ref. 39) or anterior cingulate cortex.^38^

In line with our hypotheses GSDs were nonuniformly increased in schizophrenia. Areas of the DLPFC/BA46, temporal and parietal cortex exhibit significantly increased, gyral–sulcal differences (Figure 3a). The anterior cingulate cortex showed decreased GSD in line with the neuropathological studies finding no measurable supragranular change.^38^ These findings in the DLPFC and anterior cingulate cortex are consistent with previously published neuropathology studies measuring layer thicknesses in schizophrenia (Figure 3b).

**Figure 4:**
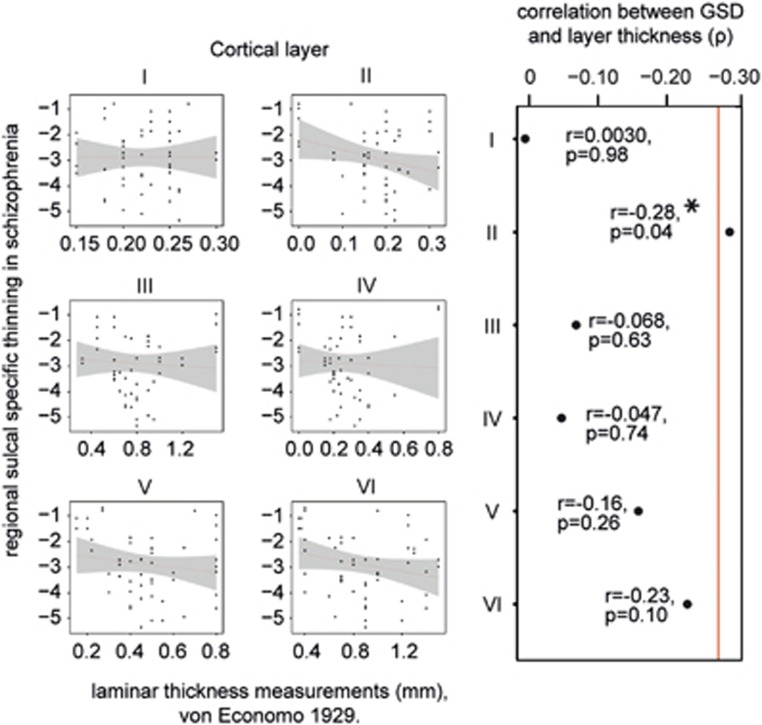
(**a**) Regional gyral–sulcal cortical thickness difference (GSD) compared with histological measurements of thickness for each cortical layer and their combined total cortical thickness, taken from von Economo.^2^ (**b**) Only layer II thickness is significantly correlated (*P*<0.05) with the regional measure of sulcal-specific thinning in schizophrenia. This did not survive correction for multiple comparisons.

The pattern of changes in gyral–sulcal differences in schizophrenia was related to neuropathological layer II thickness measurements taken from healthy individuals (*B*=−4.17, *t*=−2.11, *P*<0.05). This was not true of other cortical layers (Figure 4), suggesting that GSD was uniquely sensitive to layer II changes. This finding was not corrected for multiple comparisons. Nevertheless, the pattern of changes in schizophrenia provided further support that sulcal-specific thinning was more significant in regions with a normally thicker layer II.

### Cortical thickness gradient in visual hierarchy

**Figure 5:**
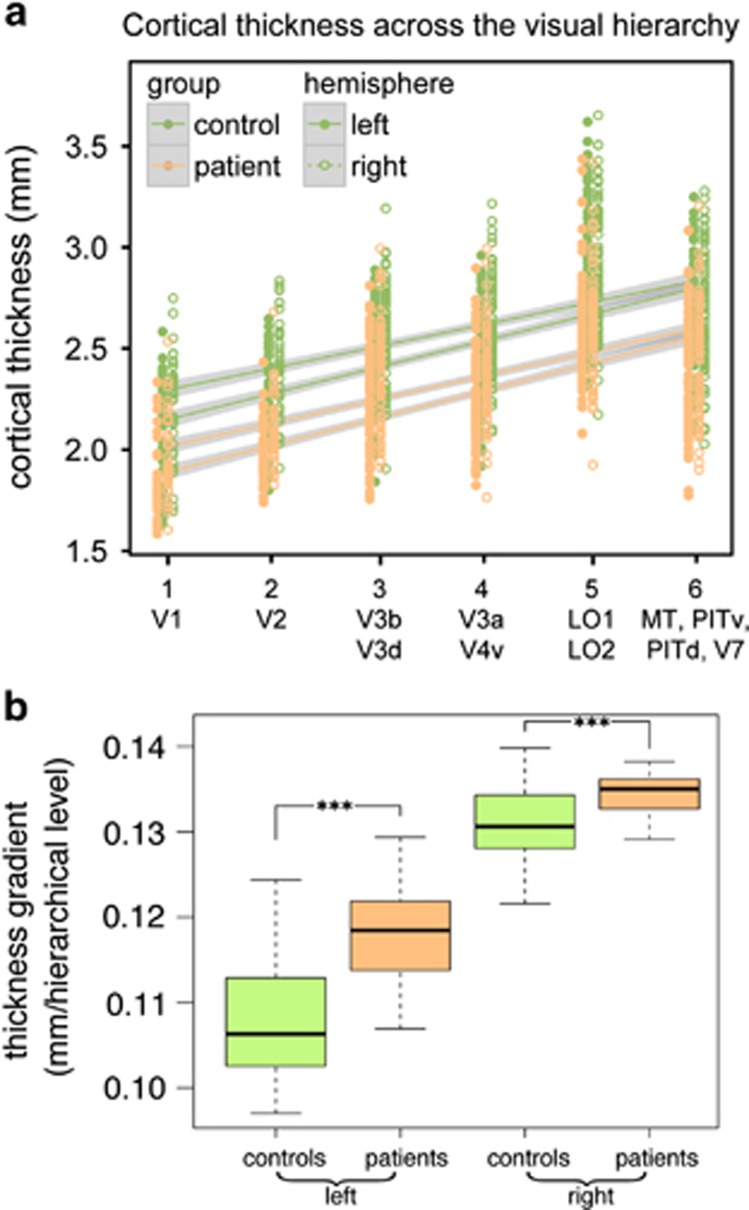
(**a**) Cortical thickness increases with visual hierarchical level in both controls and patients with schizophrenia (*P*<0.0001). Visual regions are listed below their assigned hierarchical level. (**b**) The gradient of thickness against hierarchical level is steeper in patients with schizophrenia for both left and right hemispheres (*P*<0.0001). This is consistent with supragranular thinning and has implications for the balance of feedforward/feedback connectivity in sensory regions.

Cortical thickness was strongly correlated with regional estimates of hierarchical level in both patients and controls (Figure 5a). However, the hierarchy-thickness gradient was significantly steeper in patients with schizophrenia than in healthy controls (*B*=0.0095, *t*=8.33, *P*<0.0001; Figure 5b). This is consistent with disproportionate supragranular thinning, as these layers are more prominent lower in a sensory hierarchy. There was also a significant hemisphere (*B*=0.023, *t*=27.6, *P*<0.0001) and hemisphere by diagnosis interaction (*B*=−0.006, *t*=−5.19, *P*<0.0001). There are interhemispheric asymmetries in the visual parcellation scheme used, which give rise to an apparent steepening of thickness gradient.^26^ There was no effect of sex, age or FSIQ. There was no significant effect of PANSS (*P*=0.94), medication (*P*=0.86) or illness duration (*P*=0.91) on gradients of cortical thickness within the patient group.

## Discussion

*In vivo* structural MRI measures cannot currently resolve cortical layers and therefore our ability to interpret morphological changes in terms of underlying pathological processes affecting different cortical layers is limited. In this paper we sought to develop surrogate markers of cortical structure that are sensitive to supragranular layer-specific changes. These markers are based on predictable patterns of cytoarchitecture elucidated by post-mortem studies, which have indicated that laminar thicknesses have a close relationship with macroscale structure.^2, 20^ We were therefore able to identify four morphological changes consistent with supragranular thinning, namely (i) disproportionate gyral–sulcal thinning, (ii) disproportionate alterations in gyral–sulcal pial surface intrinsic curvature, (iii) a region-specific pattern of sulcal thinning and (iv) a steeper gradient of thickness across the visual hierarchy. We tested these interlinked markers on existing data from people with schizophrenia and matched controls, accounting for the effects of medication, illness duration and severity, as well as age, sex and FSIQ. Taken together, our markers indicated that supragranular changes are present *in vivo* in patients with schizophrenia and support the evidence from functional and histological modalities that some of the deficits associated with schizophrenia may have their origin in upper cortical layer pathology.^6, 9, 40^ Moreover these measures can be applied more generally as *in vivo* structural markers of layer-specific change.

In this experiment, we adopted schizophrenia as a proof-of-concept case predicated on evidence from a wide range of neuropathology studies that identify changes to the supragranular cortical layers in the disease. In each case, our methods were in agreement with pathological evidence. For example, markers for supragranular thinning were found in the DLPFC (BA46)^13^ but not in the anterior cingulate cortex (BA24)^38^ in line with our findings of regional variation of sulcal-specific thinning.

As a marker of supragranular cortical pathology in schizophrenia, sulcal-specific thinning has further implications for understanding the development of schizophrenia. Schizophrenia commonly manifests during adolescence and is thought to relate to structural changes over this period.^41^ In particular, cortical thinning, which is nonuniform in healthy neurodevelopment, demonstrates increased thinning in sulci compared with gyri.^42^ Histologically, much of layer II merges with layer III of the cortex between late childhood and adulthood.^1^ If healthy adolescent development involves thinning and pruning of dendrites in layers II and III,^43^ for which sulcal thinning is a marker, then excessive sulcal thinning in schizophrenia^44^ might be a result of dysregulation of this normal developmental process in adolescents. Indeed, sulcal thinning in certain cortical regions has been shown to be a vulnerability indicator in schizophrenia.^45^

The fact that our observations suggest widespread supragranular changes is in keeping with the prevailing view that the range of functional changes in schizophrenia is diffuse in origin. Our fourth observation—of a steeper thickness gradient in the visual hierarchy in schizophrenia—may be interpreted in functional terms, notably with respect to the balance between top-down and bottom-up processing, which is a key part of the predictive coding model of schizophrenia.^46^ This model hypothesizes a widespread disruption, whereby the comparison between feedback predictions and feedforward sensory input to create a prediction error is perturbed. Across the cortex this comparison is made in the supragranular layers II/III (ref. 5), and multiple lines of evidence suggest that this supragranular function is disrupted in schizophrenia. Prediction errors are communicated at gamma-range oscillations,^47, 48, 49^ which are widely disrupted in schizophrenia.^50^ Moreover patients fail to modulate their response from unpredictable to predictable stimuli,^51, 52, 53^ suggesting differing feedback signals are failing to modulate prediction error signaling. Our final result demonstrates that the normal progressively changing structure of the cortex within a sensory hierarchy is disrupted in schizophrenia. Cortical hierarchies show progressive changes in cytoarchitecture and thickness, reflecting a shifting balance between feedforward and feedback connectivity.^26^ A steepening visual hierarchy suggests a shift toward stronger feedback connectivity—such a shift has also been demonstrated using functional MRI.^40^ Our results therefore offer an important link between the observation of post-mortem supragranular layer pathology and widespread functional deficits in prediction error coding in schizophrenia.

*In vivo* imaging cannot yet resolve these laminar cortical changes directly, but here we have demonstrated that certain markers of such changes can be quantified at the relatively low resolution accessible to 1.5 T or 3 T MRI. Indeed, as these are four complementary and cytoarchitecturally derived markers of cortical surface morphology, they are readily quantifiable at lower field strengths and are more robust to problems with false positives that beset vertex- or voxel-wise analyses. Although we have demonstrated their applicability in schizophrenia, the methods developed here to detect laminar cortical changes may be applied generally. For example, cortical layers follow different developmental trajectories^3^ that may produce differential development of both cortical regions^54^ and of gyri and sulci.^42^ As such, the methods presented here may be adopted as useful markers of cortical development. Similarly, many other neuropsychiatric cortical pathologies exhibit a degree of laminar specificity, including Alzheimer's disease^55^ and autism.^56^ The power to identify subtle case–control differences in these diseases may potentially be increased by adopting surrogate markers of laminar specific changes.

Morphological measures in structural MRI are necessarily limited by scale. However, by using fundamental knowledge of cortical organization, we developed a series of surrogate markers of supragranular layer changes. Although the value of the methods developed here has been demonstrated in schizophrenia, they are as generally applicable as other, more conventional approaches to studying cortical morphology. Our *in vivo* structural results are consistent with neuropathological, developmental and functional evidence that schizophrenia is characterized by widespread abnormalities in the supragranular cortical layers.
