# Supplementary figures and images for: Multiple markers of cortical morphology reveal evidence of supragranular thinning in schizophrenia
Source: Transl Psychiatry. 2016 Apr 12;6(4):e780–. doi: 10.1038/tp.2016.43 (PMC4872401; doi:10.1038/tp.2016.43)

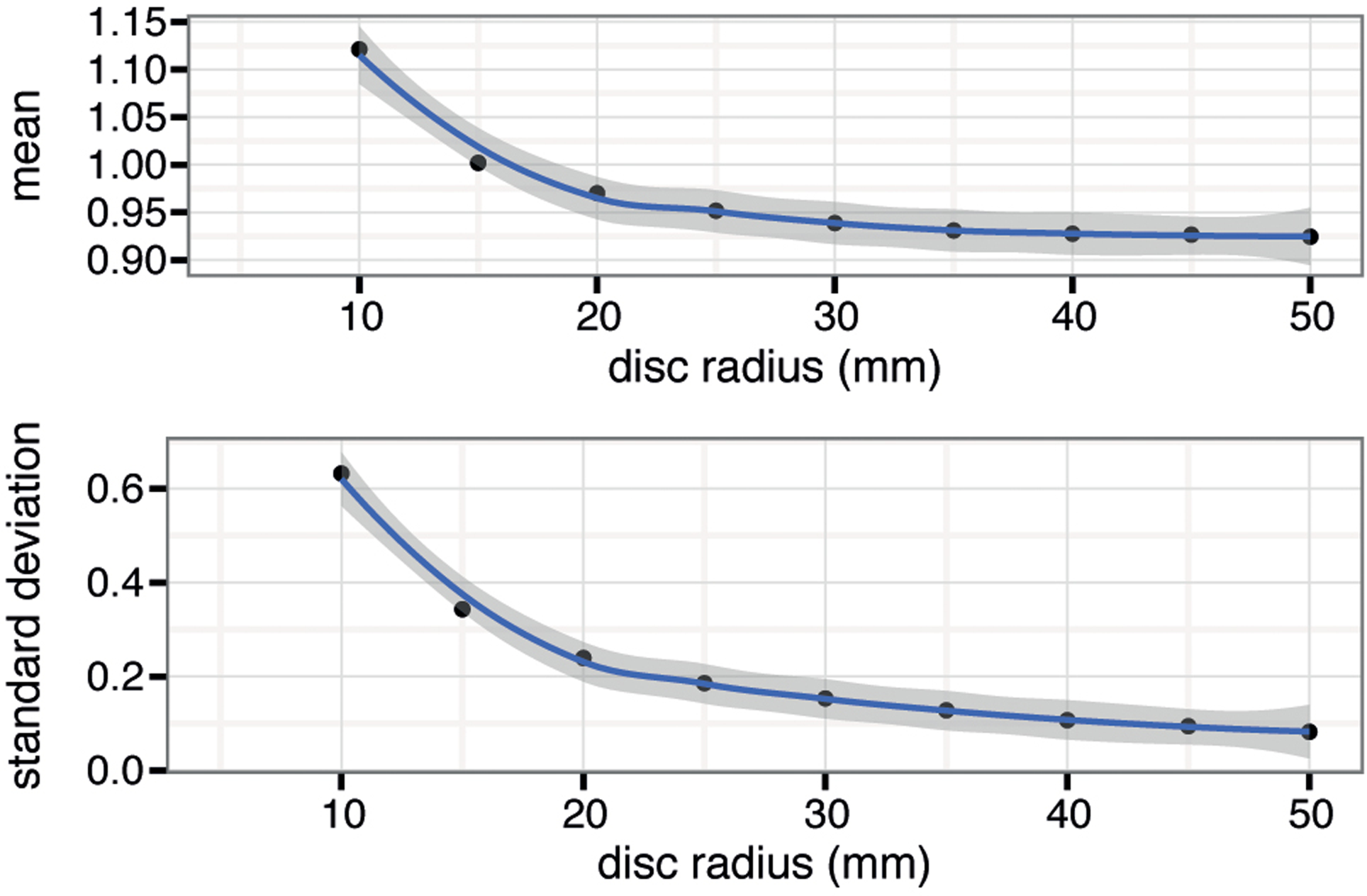

Supplement: Supplementary Figure 1 [file tp201643x1.tif]

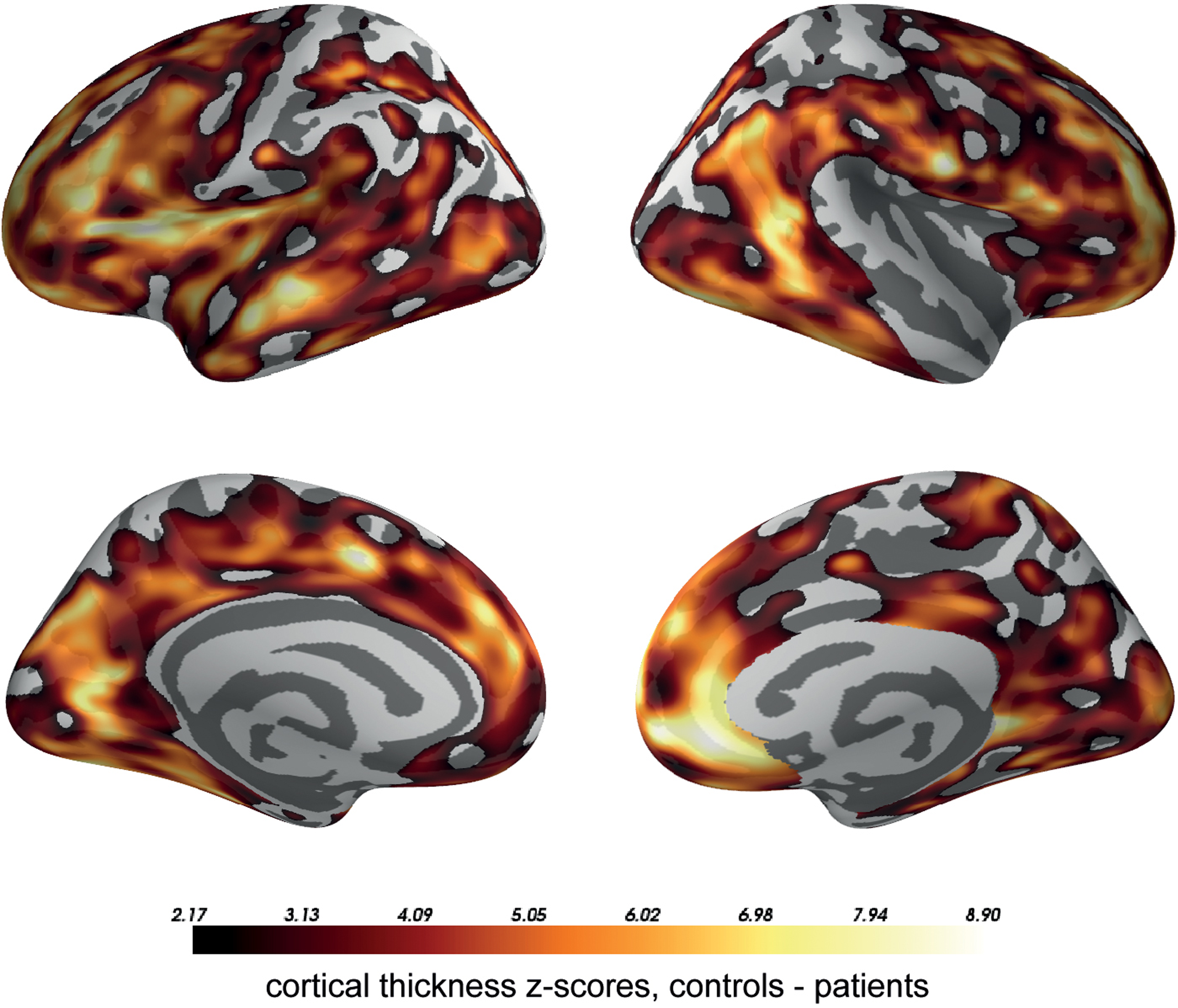

Supplement: Supplementary Figure 2 [file tp201643x2.tif]
