# Supplementary material for: Multiple markers of cortical morphology reveal evidence of supragranular thinning in schizophrenia
Source: Transl Psychiatry. 2016 Apr 12;6(4):e780–. doi: 10.1038/tp.2016.43 (PMC4872401; doi:10.1038/tp.2016.43)
Supplement: Supplementary Figure Legends [file tp201643x3.doc]

Supplementary figure legends

*Supplementary Figure 1.* The effect of increasing surface disc radius on gyral/sulcal surface area ratio sampled across the cortex of a single subject. As the disc radius increases beyond 25mm, the mean and standard deviation of this ratio plateau. Further increases in the disc radius come at a cost of local specificity of the gyral-sulcal differences (GSD) measure.

*Supplementary Figure 2.* Statistical maps showing cortical thickness differences, between controls and patients. Thickness values were smoothed at 10mm, and the linear model z-scores are thresholded at p<0.01 (FDR-corrected). Consistent with previous imaging studies of schizophrenia, there are widespread reductions in cortical thickness, including in frontal, temporal, parietal and occipital lobes.
